# Adverse effects of extra-articular corticosteroid injections: a systematic review

**DOI:** 10.1186/1471-2474-11-206

**Published:** 2010-09-13

**Authors:** Aaltien Brinks, Bart W Koes, Aloysius CW Volkers, Jan AN Verhaar, Sita MA Bierma-Zeinstra

**Affiliations:** 1 Department of General Practice Erasmus Medical Center P.O. Box 2040, 3000 CA The Netherlands; 2 Medical Library Erasmus Medical Center P.O. Box 2040, 3000 CA The Netherlands; 3 Department of Orthopaedics, Erasmus Medical Center P.O. Box 2040, 3000 CA The Netherlands

## Abstract

**Background:**

To estimate the occurrence and type of adverse effects after application of an extra-articular (soft tissue) corticosteroid injection.

**Methods:**

A systematic review of the literature was made based on a PubMed and Embase search covering the period 1956 to January 2010. Case reports were included, as were prospective and retrospective studies that reported adverse events of corticosteroid injection. All clinical trials which used extra-articular corticosteroid injections were examined. We divided the reported adverse events into major (defined as those needing intervention or not disappearing) and minor ones (transient, not requiring intervention).

**Results:**

The search yielded 87 relevant studies:44 case reports, 37 prospective studies and 6 retrospective studies. The major adverse events included osteomyelitis and protothecosis; one fatal necrotizing fasciitis; cellulitis and ecchymosis; tendon ruptures; atrophy of the plantar fat was described after injecting a neuroma; and local skin effects appeared as atrophy, hypopigmentation or as skin defect. The minor adverse events effects ranged from skin rash to flushing and disturbed menstrual pattern. Increased pain or steroid flare after injection was reported in 19 studies. After extra-articular injection, the incidence of major adverse events ranged from 0-5.8% and that of minor adverse events from 0-81%. It was not feasible to pool the risk for adverse effects due to heterogeneity of study populations and difference in interventions and variance in reporting.

**Conclusion:**

In this literature review it was difficult to accurately quantify the incidence of adverse effects after extra-articular corticosteroid injection. The reported adverse events were relatively mild, although one fatal reaction was reported.

## Background

In 1954 the first report on the effects of corticosteroids on healthy tissues appeared [1]. Local extra-articulair injections of glucocorticoid agents are currently used for rheumatic disorders including a wide spectrum of localised lesions of the tendons, enthesis, tendon sheaths, bursae, ligaments and fasciae as well as nerve compression syndromes [2]. Corticosteroid injections are frequently included as treatment option in clinical guidelines in the field of musculoskeletal disorders. Injectable corticosteroids are nowadays registered for local treatment of (rheumatic) arthritis, synovitis, bursitis, epicondylitis, tendonitis, neuromas, ganglion cysts, entrapment syndromes, fasciitis and back pain [3,4]. In 2006 Dutch pharmacists delivered 208,380 prescriptions of injectable triamcinalone, representing €2,867,000 of the €86,250,000 total prescribed medication for the musculoskeletal system (3.3%) [5] it is however not known how many of the injectable corticosteroids are given intra- or extra-articular. In a retrospective cohort study (on the five-year prognosis of trochanteric syndrome) 37% of the 164 cases were injected with corticosteroids [6]. A systematic therapeutic overview showed that 14-38% of patients with a tennis elbow in general practice were treated with corticosteroid injections [7]. In another study in general practice patients with tendosynovitis or nerve entrapment were injected with corticosteroid injection in 11% and 13% respectively [8]. Nevertheless there is only limited evidence to support the superiority of extra-articular glucocorticoid injections based on randomized trials [9]. Recently in RCT is reported efficacy of corticosteroid injections for trigger finger [10]. In addition, safety aspects of corticosteroid injections have so far not been adequately investigated by systematic reviews, except for complications associated with the use of corticosteroids in the treatment of athletic injuries [11]. Balanced decisions about healthcare interventions require evidence on harms as well as benefits [12]. Therefore, the aim of the present study was to estimate the occurrence of and describe the type of adverse effects due to extra-articular corticosteroid injections.

## Methods

### Search

The aim was to identify relevant articles describing adverse events of extra-articular corticosteroid injections. With our medical librarian (AV) we performed an extensive literature search in Pub Med and Embase covering the period 1956 until January 2010. The query was based on the definitions of terms related to adverse outcomes as stated in the Cochrane Handbook [13]. The key words and query comprised a combination of MESH terms and free-text words for injection locations (all joints, tendon, bursal, and ligamental location), with MESH terms for glucocorticosteroid products and the way of administration combined with all MESH terms and words related to adverse events. The search query is added as an additional file(see Additional file 1) In addition, the titles of references in the included articles or identified relevant reviews were checked for possibly relevant references.

Health professionals and patients can report suspicions of adverse drug reactions to the Netherlands Pharmacovigilance Centre 'Lareb'. The 'Lareb' collates adverse drug reaction data in the Netherlands; it performs this task on behalf of the Medicines Evaluation Board (MEB) [14]. The MEB is responsible for authorising and monitoring safe and effective medicinal products on the Dutch market, and shares in the responsibility for authorizing medicinal products throughout the European Union. Therefore we also contacted 'Lareb' for relevant data of adverse events reported after extra-articular corticosteroid injection.

#### Inclusion criteria

Two researchers read a share of the abstracts for inclusion in this review. Only studies that reported original patient material (e.g. case reports, case studies, cohort studies, clinical trials and case control studies) that reported on the occurrence of adverse events after intervention with local non-intra-articular corticosteroid injections were included. Studies concerning epidural injection and intramusculair injections were excluded. Because the adverse events of therapy are not always mentioned in the article abstracts, the full article of all relevant clinical trials were carefully read to find any reported adverse events [15].

### Data extraction and data syntheses

Relevant study characteristics (including authors, year of publication, size of study population, type of intervention) were extracted. In addition, the type and number of adverse events were extracted, as was the follow-up time (prospective studies) and percentage lost to follow-up (as qualitative characteristics). Where possible, the percentage of persons with adverse events was calculated separately for major adverse events (defined by us as having a lasting effect, or needing intervention) and minor adverse events (defined as transient ones not needing intervention). In the clinical trials, the frequency percentage of adverse events was calculated only for the group receiving intervention with corticosteroids. Further, a summary of the frequency of such adverse events was based on prospective studies only. Only in the case of homogenous definitions of adverse effects, interventions and study populations we did consider pooling the risk for adverse-effects. The types of major and minor adverse events were summarized separately.

The data received from 'Lareb' were also analysed separately. These data include the indication for the corticosteroid injections, gender of the patient, and route of administration. We only report here on adverse events of injections that we know for certain were applied extra-articularly.

In this review we used the terminology for adverse drug reactions noted in the Cochrane Handbook [13]. We used the term 'adverse event' for an unfavourable outcome that occurs during or after the use of a drug or other intervention but is not necessarily caused by it. 'Adverse effect' is used for an adverse event for which the causal relation between the intervention and the event is at least a reasonable possibility. Adverse drug reaction' was used for an adverse effect specific to a drug. 'Side effect' was defined by any unintended effect, adverse or beneficial, of a drug that occurs at doses normally used for treatment, and 'complications' as an adverse events or effects following surgical and other invasive interventions. In the data-extraction, however, we report the terminology used by the authors.

## Results

### Output

The search (1956 to January 2010) yielded 1,313 articles. After reading the abstracts, there were 290 possibly relevant articles on adverse effects after extra-articular injection. After studying the full-text articles and references of the included articles and relevant reviews, there were 87 relevant articles, i.e. 44 case reports, 37 prospective studies and 6 retrospective studies reporting on the adverse events of extra-articular local corticosteroid injections.

'Lareb' provided a list of reported adverse events after triamcinolone injections.

### Types of adverse events

#### Case studies

Only two articles mentioned an adverse event after a corticosteroid injection in the bursa round the hip. One of these studies reported a complication after a ten-fold higher dose was accidentally given [16], and the other reported a case of a necrotizing fasciitis after a corticosteroid injection in the trochanteric bursa - which proved to be a lethal complication [17]. Necrotizing fasciitis after corticosteroid injection for trigger finger was presented as another severe complication [18]. Six studies described hypopigmentation of the skin [19-24], and two studies described atrophy of the skin [21,23]. Atrophy of the plantar fat pad was reported after injecting an interdigital neuroma, and another group reported perilymphatic atrophy [25,26]. Atrophy of the skin and subcutaneous fat with hyperpigmentation was described in one patient after intralesional injection of a neuroma at the feet [27]. A skin defect was observed after two injections of triamcinolone injected into a hypertrophic scar [28]. Osteomyelitis of the humerus was reported after three injections with hydrocortisone for a tennis elbow [29]. Osteomyelitis of the calcaneus was reported after an injection for plantar fasciitis [30]. Localized abscess containing Staphylococcus aureus was described after injection of corticosteroid for the treatment of chronic tendinitis of the Achilles tendon [31]. Another article mentioned a sterile abscess after injecting a patient with a plantar fasciitis [32]. Protothecosis (a rare infection caused by an achlorophyllic algae) was seen in two patients after intralesional injections with corticosteroids [33]. Atypical mycobacterium soft tissue infection was reported after corticosteroid injection for de Quervain's disease [34]. An allergic reaction was reported after giving an injection to a patient who had tendonitis [35]. A tendon rupture of the hand was described after an injection into the carpal tunnel, and a tendon rupture after an injection for tennis elbow [36,37]. A delayed flexor superficiales and profundus rupture occurred after a steroid injection for trigger finger [38]. Seven weight lifters presented at the hospital with ruptured patellar tendon, they all had a history of multiple local steroid injections [39]. A rupture of the Achilles tendon associated with corticosteroid injections was reported in three studies [40-42]. Another case report described an avulsion of the calcaneal tendon after steroid injections administered because of an acute flare-up of rheumatoid arthritis [43]. One study described thirteen patients who developed 15 ruptured tendons subsequent to injection of a depository steroid in or around the tendons injected [44]. One study reported ischemia of the hand after carpal tunnel injection and one study after a corticosteroid injection for de Quervain tenosynovitis [45,46]. Nerve injury after steroid injection for carpal tunnel syndrome is described in 3 studies [47-49]. Soft tissue calcifications were reported as a complication due to adjusted materials in the solvent or due to an accumulation of insoluble steroid [50-52].

#### Prospective studies

Of the 37 prospective studies, 11 reported no adverse effects. Hypopigmentation was reported in three studies [53-55]. Atrophy was described in four studies [54,56-58]. Increased or persistent pain after injection or pain at the site of injection was described in 19 studies. Adverse events not mentioned in the case reports were flushes and disturbance in menstrual pattern [59]. Cellulitis, ecchymosis and subcutaneous nodule were three other symptoms not mentioned earlier in the case reports [55,60]. Table 1 presents information on the minor and major adverse events in the prospective studies.

**Table 1:** Summary of the included prospective studies.

*Author, Year of publication*	*Type of study, number of cases*	*Indication*	*Corticosteroid injection agent (no. of injected cases, sides)*	*Methods of reporting adverse events*	*Complications reported Major/minor Period of time*	*Follow-up (%)*
Rompe 2009 [69]	RCT (229)	GTPS	Prednisolon 25 mg/meaverin 0.5% (75)	In method section: adverse effects were recorded by the physician Results: summarised in a table	Minor: increased or radiating pain: 44%, skin irritation 3%, swelling 9%	92% after 15 months

Gunter 2004 [70]	RCT (18)	Iliotibial band friction syndrome	Methylprednisolone acetate 40 mg/lignocaine 1% (9)	In method section: side effects/adverse reactions: are reported in both intervention groups as a separate issue Results: mentioned as a separate issue	No side-effects after 7 and 14 days	100% at 2 weeks

Chao 2009 [71]	RCT (97)	Trigger thumb	Triamcenolon 10 mg (42)	In method section: no information Results: mentioned in a sentence	Minor: 2.2% had pain after 1 month	100% after 12 months

Peters 2008 [72]	RCT (50)	Trigger finger	Triamcinolone acetonide: 10, 1 or 2 injections (41)	In method section: adverse event as secondary outcome Results: mentioned as a separate issue	Minor: hot flushes 22%, steroid-flare 14.6%	82% after 12 months follow-up

Jianmongkol 2007 [73]	RCT (101)	Trigger finger, 2 types of injection therapy were compared (48/53)	Triamcinolone 10 mg/lidocaine (101)	In method section: no information Results: reported in one sentence	No complications	Follow-up 6 weeks (% lost to follow-up not mentioned)

Goldfarb 2007 [74]	RCT (154)	Trigger finger or De Quervain's tenosynovitis	Methylprednisolone acetate 40 mg/lidocaine 1%/bupivacaine 0.5% (154)	In method section: incidence of post injection pain flare was the aim of the study, no other complications are monitored Results: flare reaction mentioned as a separate issue	Minor: in 33% increase in pain score of 2 points or more (VAS scale 0-10).	81% follow-up after 1 and 6 weeks

Baumgarten 2007 [75]	RCT (59)	Trigger finger in diabetics versus non diabetics	Betamethasone 6 mg/lidocaine 1% (44)	In method section: in follow-up section: complications related to treatment were reported Results: complications reported as a separate issue	No adverse events at 6 weeks, 3 months and 1 year	98% follow-up at 12 month (range 13-41 months)

Kazuki 2006 [76]	Pros (100)	Trigger finger	Betamethasone 2.5 mg/lidocaine 1% (129 fingers)	In method section: not mentioned. Results: one sentence: no complications of steroid injections were observed	No complications after 6 months	100% follow-up after 6 months (range 1-42)

Gurcay 2009 [77]	RCT (36)	Carpal tunnel syndrome	Betamethasone 6 mg (18)	In method section: not mentioned Results: no complications or side effects to treatment were observed	No side effects	100% follow-up after 3 months

Nalamachu 2006 [78]	RCT (40)	Carpal tunnel syndrome	Methylprednisolone 40 mg/lidocaine 1% (20)	In method section: adverse events were classified according to MedDRA and the incidence of treatment emergent events was summarized Results: adverse events were described	Minor: numbness (5%), local pain (5%), tingling in hands at 4 weeks (5%)	85% follow-up after 4 weeks

Dammers 2005 [79]	RCT (132)	Carpal tunnel syndrome	Methylprednisolone 20 mg (45), 40 mg (43), 60 mg (44) with lidocaine 10 mg	In method section: not mentioned Results: no side effects were recorded	No side-effects after 1 and 12 months	97% follow-up after 12 months

Hui 2005 [60]	RCT (50)	Carpal tunnel syndrome	Methylprednisolon 15 mg (25)	In method section: surgical complications are assessed after one week, no other adverse events mentioned. Results: one patient with cellulitis is reported and four patients with pain at the injection side	Minor: pain at injection side 16% Major: cellulitis 4%	100% at 6 and 20 weeks

Wong 2005 [80]	RCT (40)	Carpal tunnel syndrome	Methylprednisolone 15 mg single dose (20) or double dose (20)	In method section: any side effects were recorded at 8, 24 and 40 weeks Results: reported as a sentence at the end of the result section	Minor: local pain (30% in 20 mg group and 10% in 20 mg group)	100% follow-up at 8 weeks

Agarwal 2005 [81]	Pros (48)	Carpal tunnel syndrome	Methylprednisolone acetate 40 mg/xylocaine 2% (67 hands)	In method section: not mentioned Results: at the end of the result section adverse effects were mentioned	Minor: mild discoloration of the skin over the injection site (6%)	100% follow-up after 3 months, 78% after 12 months

Ly-Pen 2005 [82]	RCT (163)	Carpal tunnel syndrome	Paramethasone acetonide 20 mg (82, 69 wrists required second injection)	In method section: not mentioned Results: safety and tolerability was a separate chapter	No relevant side-effects	79.5% follow-up at 12 months

Sevim 2004 [83]	RCT (120)	Carpal tunnel syndrome	Betamethasone 6 mg. (60)	In method section: not mentioned Results: complications and side effects are described	Minor: moderate pain lasting less than 24 hours after injection (3.4%), haematoma (1.7%)	90% follow-up at 11 months follow-up (range 9 to 14 months)

Armstrong 2004 [84]	RCT (81)	Carpal tunnel syndrome	Betamethasone 6 mg/lidocaine 1% (43 with a total of 364 injections)	In method section: side effects and complications are recorded Results: adverse effects described	Minor: severe pain after injection (5%), acute transient sympathetic reaction after injection (2%)	89% follow-up after 18 months

Wong 2001 [85]	RCT (62)	Carpal tunnel syndrome	Methylprednisolone 15 mg (30)	In method section: any side effects were recorded by telephone interview Results: summarized in a table	Minor: injection pain (6.7%)	100% after 12 weeks

Kalaci 2009 [64]	RCT (100)	Plantar fasciitis	Triamcinolone 20 mg (50)	In method section: not mentioned Results: description of the side effects not found	No side effects or complications All of the patients found the injection painful	100% after 6 months

Porter 2005 [86]	RCT (132)	Plantar fasciopathy	Betamethason 5.7 mg/lignocaine 1% (64)	In method section: patients were asked to report any possible side effects at 3 and 12 months Results: no infections or rupture are found, description of the side effects	Minor: post-injection pain (12.5%) that required analgesia and/or ice application	95% follow-up at 12 months

Genc 2005 [87]	Pros (30)	Plantar fasciitis	Methylprednisolone 20 mg/prolocaine 2% (47 heels)	In method section: ultrasonografy measurement of the facia at 1 and 6 months Results: reported as one sentence	No rupture observed	100% follow-up at 6 months

Lindenhovius 2008 [88]	RCT (64)	Lateral elbow pain	Dexamethasone 4 mg/lidocaine 1% (31)	In method section: not mentioned Results: adverse events are described	Minor: discoloration of skin 3.2% increased elbow pain 3.2%	77% after 1 and 6 months

Tonks 2007 [53]	RCT (48)	Epicondylitis lateralis	Triamcinolone acetonide 10 mg/lignocaine 2% (24)	In method section: complications of treatment were one of the outcome measurements Result section: complications are described	Major: skin depigmentation and atrophy in 4% after 7 weeks	77% follow-up at 7 weeks

Bisset 2006 [54]	RCT (198)	Tennis elbow	Triamcinolone 10 mg/lidocaine 1% (65)	In method section: not mentioned Results: side effects were mentioned in a separate section	Minor: pain (18.5%). Major: loss of skin pigment (3%), atrophy of subcutaneous tissue (1.5%)	100% follow-up in injection group at 12 months

Wang 2003 [89]	Pros (94)	Hand and elbow injections	Betamethasone/lidocaine 1%	In method section: registration of pain levels after injection of corticosteroid to hand and elbow was the aim of the study, no other side effects were recorded Results: post injection pain is shown in table and list	Minor: 50% increased post-injection pain during 1.2 days	71% follow-up at 5 days

Smidt 2002 [90]	RCT (185)	Epicondylitis lateralis	Triamcinolone acetonide 10 mg/lidocaine (62)	In method section: details of any adverse effects were reported on standardised forms Results: adverse effects summarized in a table	Minor: facial flush (3%), skin irritation (5%), red swollen elbow (3%), change of skin colour (11%), other not specified side-effects (13%)	96% follow-up at 52 weeks

Jensen 2001 [91]	RCT (30)	Tennis elbow	Methylprednisolone 20 mg/lidocaine 1% (16)	In method section: daily pain registration for six weeks Results: described in result section	Minor: pain increase after injection (81%)	100% follow-up 6 weeks

Hay 1999 [57]	RCT (164)	Tennis elbow	Methylprednisolone 20 mg/lignocaine (51)	In method section: complications of treatment is one of the secondary outcome Results: described in a separate section side effects	Major: local skin atrophy in the overall group (3 of 111), one with steroids (1.9%)	100% follow-up at 12 months

Stahl 1997 [92]	RCT (58)	Medial epicondylitis	Methylprednisolone 40 mg/lidocaine (30)	In method section: interviews and physical examination for possible local complications Results: complications are reported in a separate part	Major: non reported Minor: facial flushing in one female patient	100%follow-up 12 months

Verhaar 1995 [93]	RCT (106)	Tennis elbow	Triamcinolone 1% (53)	In method section: side effects not specified Results: no infection or skin hypopigmentation	No side effects in the injection group	100% follow up after 12 months

Price 1991 [58]	RCT (145)	Tennis elbow	Triamcinolone 10 mg/lignocaine 1% or Hydrocortisone 25 mg/lignocaine 1% compared with lignocaine 1% or with Triamcinolone 20 mg. Second study Triamcinolone 10 mg versus 20 mg	In method section: severe post-injection pain and skin atrophy were noted Results: table with the adverse effects	Minor: post-injection pain (11%-58%). Major: skin atrophy (17%-40%)	Follow-up at 24 weeks (% lost to follow-up not clear)

Jirarattanaphochai 2004 [55]	RCT (160)	De Quervain's tenosynovitis	Triamcinolone acetonide 10 mg (100)	In method section: the adverse events reported at 3 weeks, 6 and 12 months are secondary outcome measurements Results: adverse effects are mentioned in a table	Minor: post-injection pain (13%), subcutaneous nodule (2.5%), ecchymosis (1.3%). Major: skin hypopigmentation (1.3%)	100% follow-up, 3% lost between 6 and 12 months

Avci 2002 [94]	CT (19)	Pregnant or lactating women with De Quervain's tenosynovitis	Methylprednisolone 10 mg (10)	In method section: not mentioned Result section: not specified	No side effects or local complications of corticosteroid injection were noted	100% follow (range 9-17 months)

Anderson 1991[95]	Pros (56)	De Quervain tenosynovitis	Methylprednisolone acetate 40 mg	In method section: adverse reaction were recorded, particularly signs of atrophy Result: adverse reactions are summarised in a table	Minor; pain 18%, pain, swelling, heat 5% ecchymosis 9% temporary radial nerve paresthesia 2% vasovagal reaction 2% Major: subcutaneous fat atrophy 16%	95% follow-up at 4.2 years

Crawford 1999 [96]	RCT (106)	Heel pain	Methylprednisolone 25 mg (53)	In methods and results sections: not mentioned	No side-effects reported	52% follow-up after 6 months

Capasso 1997 [97]	RCT (116)	Patellar tendopathy	Methylprednisolone 40 mg/lignocaine (39)	In method section: not mentioned Results: acceptability of treatment is separately discussed in a chapter	Minor: burning sensation (10.3%) injection pain (5.1%)	82% follow-up after 12 months

Mens 1998 [59]	Pros (77 ♀)	Musculo-skeletal disease	Triamcinolone acetate intra-articular (46) and extra-articular (24)	Method section: patients were asked to report appearance of flushing and any abnormality of the menstrual pattern Results: shown in a table	Disturbance in menstruation at 6 weeks (50.6%), flushes (28.6%)	100% follow-up after 6 weeks

RCT: randomized controlled trial, CT: controlled trial, Pros: prospective clinical study

#### Retrospective studies

In one retrospective study septic bursitis was described after corticosteroid injection in traumatic olecranon bursitis [61]. Tachon's syndrome (subacute back pain and/or thoracic pain following local injections of corticosteroids) was reported in one study [62]. Table 2 presents information on the adverse events in the retrospective studies.

**Table 2:** Summary of the included retrospective studies

Author, Year of publication	Type of study	Indication	Corticosteroid used	Complication (number of cases)
Berthelot 2004 [62]	Questionnaire sent to 500 rheumatologist	Different rheumatologic diseases	Cortivazol Hydrocortisone Betamethasone Paramethasone	Tachon's syndrome (n = 318) *

Cill 2004 [98]	48 cases	Achilles tendinopathy	Triamcinolone 10 mg and 20 mg with bupivacaine 0.25%	No major complications, 1 patient (2%) reported purple skin dicoloration

Bjorkman 2004 [99]	27 cases	Rupture of the tendon extensor pollicis longus	2 oral corticosteroids and 2 local corticosteroid injections	Rupture of the tendon extensor pollicis longus n = 4 associated with use of corticosteroids

Acevedo 1998 [100]	765 cases	Plantar fasciitis	Triamcinolone 40 mg (122)	Plantar fascia rupture (n = 44) **

Astrom 1998 [101]	298 cases	Achilles tendinopathy	Unknown	Preoperative steroid injection was predictive of a partial rupture***

Weinstein 1984 [61]	Follow-up of 47 cases	Traumatic olecranon bursitis	25 patients received Triamcinolone 20 mg after aspiration	septic bursitis (9%) skin atrophy (25%) chronic pain (28%)

* 1 event per 8,000 injections

** 44 of the 51 plantar fascia ruptures were associated with corticosteroids injection

***Odds ratio 2.0 (CI 1.3-9.8)

#### Lareb Institute

The following adverse events were registered by the 'Lareb' institute following extra-articular indications: after corticosteroid injection for bursitis trochanterica flushing was reported, after injection for tennis elbow, rash, menstrual disorder, and skin depigmentation and in one patient dyspnoea and eyelid ptosis were reported. In one patient hallucination, increased intracranial and intraocular pressure, and paresis occurred after corticosteroid injection for a calcaneal spur. In another patient, after corticosteroid injection for carpal tunnel syndrome hirsutism, nail changes and vaginal hemorrhage were reported. After injection for trigger finger an allergic skin reaction was observed. Reported adverse events after corticosteroid injections for tendonitis were: anaphylactic reaction in one patient, erythema and skin atrophy in another, and rash and tendon disorder in the third patient.

##### Frequency of adverse events

Due to the heterogeneity of the study populations, the type of interventions, the uncertain causality of the reported reaction with the administered corticosteroid injection and the impossibility to count risk differences in all the studies, we refrained from pooling the risk for adverse-effects. Minor adverse events were:

- pain after injection with a frequency ranging from 3.4-81%

- numbness and tingling in hands was reported in one study on CTS patients in 5% of the cases

- mild discoloration of the skin over the site of injection in three studies in 3.2%, 6% and 11.2%, respectively

- disturbance in menstruation in one study in 50.6% of the patients, and flushes in 3 studies with a frequency of 3.2%, 22% and 28.6%, respectively

-transient sympathetic reaction in one study with a frequency of 2%

- ecchymosis in one study with a frequency of 1.3%

Major adverse events in the prospective studies were:

- skin depigmentation reported in 3 studies with a frequency ranging from 1.3-4%

- atrophy was mentioned in 5 studies with a frequency ranging from 1.5-40%

- cellulitis was reported in one study in 4% of the patients

## Discussion

In this review, reported dermal adverse events of local corticosteroid injections were irritation, change of skin colour, skin and perilymphatic atrophy, soft tissue calcification, skin defect, hypopigmentation, sterile abscess, ecchymosis, and allergic rash. The infectious adverse events were cellulites, localized abscess, septic bursitis, atypical mycobacterium infection, necrotizing fasciitis, and protothecosis. Local adverse events included local pain, tingling or numbness in hands, local neural damage and tendon rupture. Systemic adverse events included allergic reactions, facial flush and disturbance in menstrual pattern.

Edwards and Aronson defined an adverse drug reaction as "*an appreciably harmful or unpleasant reaction, resulting from an intervention related to the use of a medicinal product, which predicts hazard from future administration and warrants prevention or specific treatment, or alteration of the dosage regimen, or withdrawal of the product*" [63]. According to the WHO they classify adverse drug reactions into six types: dose-related, non dose-related, dose-related and time-related, time-related, withdrawal, and failure of therapy. In the present review we were unable to categorize the adverse drug reaction in this way. We neither were able to judge the causal relation between the reported reactions and the administered drug, so we are about speaking of adverse events rather than adverse drug reactions. In addition, in table 1 we reported the terminology as described in the individual articles, because it was not always clear which classification system they used. Although the adverse events reported in our review are 'miscellaneous' ones, we think that these types of adverse events (occurring after a regular dose of extra-articular corticosteroid injections) can be divided in systemic adverse events and local adverse events. The systemic adverse events can be divided into allergic reactions (IgE mediated) or other hypersensitivity reactions, disturbance in menstruation, flushes and Tachon's syndrome and systemic infection. The local effects consisted of local pain, degeneration, atrophy and change in skin colour, local infection, impact on collagen metabolism expressed as tendon ruptures, and perilymphatic atrophy. Depending on the place where the injection is administered, adverse events can manifest, for example, injections for plantar fasciitis are almost painful [64]. Injections in general can cause substantial adverse effects. For example, Nicolau's syndrome (livedoid dermatitis secondary to acute arterial thrombosis after injection in a blood vessel) has been described after an intra-articular corticosteroid injection [65]. Such an adverse event would be extremely rare after injection in a bursa or other superficial structures. The venous counterpart, known as Tachon's syndrome (subacute back pain and/or thoracic pain following local injections of corticosteroids), was reported in a retrospective study [62]. In general it obvious that adverse events associated with corticosteroid injection can be minimised by ensuring appropriate injecting procedures are followed by a well-trained practitioner. Neural damage after injecting CTS might be avoided by proper injection technique [66]. In this review we divided the adverse events into minor ones (the harm was temporary) and major ones (the adverse event needed intervention or was not transient). This clinical categorization, although not approved by the WHO or FDA, might help to make a more balanced decision regarding the (possible) harm of an injection with corticosteroids for extra articular use. In addition, it can be easily explained to patients.

The Cochrane Collaboration provides guidance from the Adverse Effects Subgroup of the Non-randomized Studies Methods Group [13]. An appendix provides information on adverse effects, advice and tips about the search strategy and the type of studies to be included. However, we failed to find all the relevant articles with the search strategy advised by the Cochrane Collaboration and had to expand the search strategy. In our review, we did not use an overall quality assessment. We did describe however, the methods of reporting adverse events for each prospective study, the duration of follow-up, and the percentage lost to follow-up. The drawback of our study is that we could not assess the risk of bias. Clinical trials, cohort studies and case studies have their own risk of bias [13]. The limitations of the case reports are that there is uncertainty as to the adverse event was caused by the corticosteroid injection. Similarly, the lack of a control group in the prospective study on reporting specified menstruation disorders afterwards cannot prove the causal relationship [59]. If we assume that the internal validity for assessing adverse events in RCT at least should be based on the percentage available for follow-up (i.e. 80% or more) and systematic registration of adverse events and a comparison against a control group, then using these criteria less than half of the prospective studies in this review were of inferior quality.

Some RCTs assess smaller numbers of patients thus decreasing the chance of detecting a rare adverse event. Moreover, a part of the RCTs cover a relatively short study period thus precluding the identification of delayed or prolonged, and generally have highly specific inclusion/exclusion criteria that may imply that the results cannot be generalized to other populations.

Therefore, the assessment of safety needs to cover not only RCTs but also explore other sources such as, for example, post-marketing surveillance studies, spontaneous reporting schemes, and epidemiological studies. Systematic reviews on the safety of therapeutic interventions should preferably combine data from various types of studies [67].

In prospective studies, adverse effects attributed to the specific intervention should preferably be estimated by risk ratios, where the risk for adverse effects in the intervention group is compared with that for those who did not receive the intervention. In the present review, because all subjects included in the prospective studies received the intervention, only the percentage of adverse effects could be estimated. For this reason, in the RCTs we estimated the percentage of adverse effects for the intervention group only and did not compare these data with the control group. However, because the types of adverse event reported in these RCTs were highly intervention-specific we do not expect an overestimation of the adverse effects. In fact, based on the inadequate/lack of systematic registration in the included studies, we suspect there may even be an underestimation of the adverse-effects. Therefore we advocate that future RCTs and prospective studies should report on adverse events following the recommendations in the CONSORT guidelines [68].

## Conclusion

In this literature review it was difficult to accurately quantify the incidence of adverse effects after extra-articular corticosteroid injection. Although one fatal adverse event after an extra-articular corticosteroid injection was reported, extra-articular corticosteroid injections are regularly administered worldwide. In the present review the incidence of major adverse events (according to our definition) was up to 5.8%, ranging from depigmentation and atrophy of the skin to cellulitis; generally speaking these adverse effects could perhaps be classified as 'relatively mild'. Based on these data the administration of extra-articular corticosteroid injections seems to be a 'relatively safe' intervention.

## Competing interests

The authors declare that they have no competing interests.

## Authors' contributions

AB designed the search strategy and read the abstracts and wrote the manuscript, AV carried out the search strategy in Pubmed and Embase, SB participated in its design and coordination and read a part of the abstracts and have been involved in drafting the manuscript. BK and JV participated in its design and coordination.

All authors have read and approved the final manuscript.

## Pre-publication history

The pre-publication history for this paper can be accessed here:

http://www.biomedcentral.com/1471-2474/11/206/prepub

## Supplementary Material

Additional file 1 **Search strategy in Pubmed and Embase**. Word DOC displaying search strategy in Pubmed and Embase. Click here for file
