# Supplementary material for: Adverse effects of extra-articular corticosteroid injections: a systematic review
Source: BMC Musculoskelet Disord. 2010 Sep 13;11:206. doi: 10.1186/1471-2474-11-206 (PMC2945953; doi:10.1186/1471-2474-11-206)
Supplement: Additional file 1 — Search strategy in Pubmed and Embase. Word DOC displaying search strategy in Pubmed and Embase. [file 1471-2474-11-206-S1.DOC]

(((((joint OR joints OR joint* OR tendon OR tendinitis OR bursitis OR bursa OR ligament OR ligaments OR ligaments* OR intraarticular OR shoulder OR shoulders OR knee OR knees OR foot OR elbow OR elbows OR wrist OR wrists OR hip OR hips OR heel OR heels)))

(injections[mesh] OR injection*[tw]) AND (glucocorticoids[mesh] OR glucocorticoid*[tw] OR corticosteroid*[tw]) AND (adverse effects[sh] OR adverse[tw] OR side effect*[tw] OR safety[tw] OR tolera*[tw] OR poison*[tw] OR toxic*[tw] OR chemically induced[sh] OR chemically induced[tw] OR contraindicat*[tw] OR contra-indicat*[tw] OR complicat*[tw])

(extra-articul*[tw] OR extraarticul*[tw]) AND (injections[mesh] OR injection*[tw]) AND (glucocorticoids[mesh] OR glucocorticoid*[tw] OR corticosteroid*[tw]) AND (adverse effects[sh] OR adverse[tw] OR side effect*[tw] OR safety[tw] OR tolera*[tw] OR poison*[tw] OR toxic*[tw] OR chemically induced[sh] OR chemically induced[tw] OR contraindicat*[tw] OR contra-indicat*[tw] OR complicat*[tw])

PubMed

(tendons[mesh] OR tendo*[tw] OR teno[tw] OR tendinopathy[mesh] OR tendin*[tw] OR enthesiti*[tw] OR bursa*[tw] OR bursitis[mesh] OR bursit*[tw] OR periarthrit*[tw] OR ligaments[mesh] OR ligament*[tw] OR knee[mesh] OR knee*[tw] OR foot[tw] OR feet[tw] OR foot diseases[mesh] OR fasciiti*[tw] OR heel*[tw] OR epicondyl*[tw] OR elbow*[tw] OR wrist*[tw] OR hip[tw] OR hips[tw] OR extra-articul*[tw] OR extraarticul*[tw]) AND (injections[mesh] OR injection*[tw]) AND (adrenal cortex hormones[mesh:noexp] OR glucocorticoids[mesh] OR hydroxycorticosteroids[mesh] OR glucocorticoid*[tw] OR hydroxyglucocorticosteroid*[tw] OR corticosteroid*[tw]) AND (adverse effects[sh] OR adverse[tw] OR side effect*[tw] OR safety[tw] OR tolera*[tw] OR poison*[tw] OR toxic*[tw] OR chemically induced[sh] OR chemically induced[tw] OR contraindicat*[tw] OR contra-indicat*[tw] OR complicat*[tw])

EMbase

(tendon/syn OR tendo*:ti,ab,de OR teno:ti,ab,de OR periarthritis/exp OR tendinitis/syn OR tendin*:ti,ab,de OR enthesiti*:ti,ab,de OR bursa*:ti,ab,de OR bursit*:ti,ab,de OR periarthrit*:ti,ab,de OR ligament/syn OR ligament*:ti,ab,de OR knee*:ti,ab,de OR foot:ti,ab,de OR feet:ti,ab,de OR fasciiti*:ti,ab,de OR heel*:ti,ab,de OR epicondyl*:ti,ab,de OR elbow*:ti,ab,de OR wrist*:ti,ab,de OR hip:ti,ab,de OR hips:ti,ab,de OR (extra NEAR/1 articul*):ti,ab,de OR extraarticul*:ti,ab,de) AND (injection/syn OR injection*:ti,ab,de) AND (glucocorticoid/syn OR glucocorticoid*:ti,ab,de OR corticosteroid*:ti,ab,de) AND ('adverse drug reaction'/syn OR 'adverse effect':lnk OR adverse:ti,ab,de OR 'side effect':ti,ab,de OR 'side effects':ti,ab,de OR safety:ti,ab,de OR tolera*:ti,ab,de OR poison*:ti,ab,de OR toxic*:ti,ab,de OR 'chemically induced':ti,ab,de OR contraindicat*:ti,ab,de OR (contra NEAR/1 indicat*):ti,ab,de OR complicat*:ti,ab,de)
